# Trajectory Tracking Controller for Quadrotor by Continual Reinforcement Learning in Wind-Disturbed Environment

**DOI:** 10.3390/s25164895

**Published:** 2025-08-08

**Authors:** Yanhui Liu, Lina Hao, Shuopeng Wang, Xu Wang

**Affiliations:** School of Mechanical Engineering and Automation, Northeastern University, Shenyang 110819, China

**Keywords:** quadrotor, deep reinforcement learning, continual learning, trajectory tracking control

## Abstract

The extensive deployment of quadrotors in complex environmental missions has revealed a critical challenge: degradation of trajectory tracking accuracy due to time-varying wind disturbances. Conventional model-based controllers struggle to adapt to nonlinear wind field dynamics, while data-driven approaches often suffer from catastrophic forgetting that compromises environmental adaptability. This paper proposes a reinforcement learning framework with continual adaptation capabilities to enhance robust tracking performance for quadrotors operating in dynamic wind fields. We develop a continual reinforcement learning framework integrating continual backpropagation algorithms with reinforcement learning. Initially, a foundation model is trained in wind-free conditions. When wind disturbance intensity undergoes gradual variations, a neuron utility assessment mechanism dynamically resets inefficient neurons to maintain network plasticity. Concurrently, a multi-objective reward function is designed to improve both training precision and efficiency. The Gazebo/PX4 simulation platform was utilized to validate the wind disturbance stepwise growth and stochastic variations. This approach demonstrated a reduction in the root mean square error of trajectory tracking when compared to the standard PPO algorithm. The proposed framework resolves the plasticity loss problem in deep reinforcement learning through structured neuron resetting, significantly enhancing the continual adaptation capabilities of quadrotors in dynamic wind fields.

## 1. Introduction

With the rapid advancement of the unmanned aerial vehicle (UAV) domain, the quadrotor—owing to its distinctive characteristics—has been extensively employed in various UAV control systems [1,2,3,4,5]. The simple structure, high stability, strong maneuverability, rapid response, and exceptional payload adaptability of the system allow for high-precision execution of tasks, including aerial hovering and trajectory tracking [6,7,8,9]. However, in practical applications, quadrotors are often confronted with challenges, including the difficulty of accurately modeling wind dynamics, uncertainty in environmental parameters, and inherently nonlinear dynamical equations [10,11]. Thus, it is both critical and urgent to deepen our understanding of UAV adaptability, robustness, and intelligence under environmental disturbances.

Outdoor wind disturbances represent a significant factor affecting the stability and performance of quadrotor control systems. To achieve precise trajectory tracking of a quadrotor in the presence of such disturbances, existing control approaches under complex environmental conditions can be broadly categorized into model-based and data-driven strategies. Model-based methods explicitly incorporate aerodynamic disturbance terms into the quadrotor’s dynamical equations and design disturbance-rejection controllers to enhance tracking accuracy [12,13]. In [14], a robust H_∞_ guaranteed cost controller is developed for quadrotors, concurrently addressing model uncertainties and external disturbances to effectively attenuate perturbation effects. In [15], a sliding-mode controller is combined with a fixed-time disturbance observer to design a controller for the position–attitude system, thereby handling time-varying wind disturbances in trajectory tracking. In [16], a model predictive control-based position controller is integrated with an SO(3)-based nonlinear robust attitude control law to counteract external disturbances. Model-based controllers largely depend on accurate dynamical models of wind disturbances and can deliver stable and reliable performance under low uncertainties. Nonetheless, these controllers often exhibit limited adaptability when handling complex nonlinear and multivariable coupling effects, and their parameter tuning presents significant difficulties.

Compared to model-based control strategies, data-driven approaches demonstrate enhanced adaptive capabilities when addressing complex nonlinear, time-varying, or unknown disturbances [17,18,19]. Regarding data-driven state observers for quadrotors, ref. [20] employed Koopman operator theory to construct dynamics in two distinct environments and integrated them with an MPC controller for trajectory tracking. Ref. [21] proposed a reinforcement learning (RL) framework incorporating external force compensation and a disturbance observer to attenuate wind gust effects during trajectory tracking. In [22], a novel wind perturbation estimator utilizing neural networks and cascaded Lyapunov functions was used to derive a full backstepping controller for quadrotor trajectory tracking. Beyond observer design, deep reinforcement learning (DRL) methods enable direct policy learning through reward function design, allowing quadrotors to interact with wind-disturbed environments. In [23], an RL-based controller was trained to directly generate desired three-axis Euler angles and throttle commands for disturbance rejection. Ref. [24] proposes a distributed architecture utilizing multi-agent reinforcement learning to reduce user perception delay and minimize UAV energy consumption. Ref. [25] integrated policy relief and significance weighting into incremental DRL to enhance control accuracy in dynamic environments. In [26], dual-critic neural networks supplant the conventional actor–critic framework to approximate solutions to Hamilton–Jacobi–Bellman equations for disturbance-robust quadrotor tracking. However, such data-driven methods typically employ static neural networks with frozen weights post-training. This approach relies on wind-condition-specific datasets during offline training, limiting generalization capability beyond the training distribution. Moreover, when environmental dynamics drift, conventional online fine-tuning strategies are highly susceptible to catastrophic forgetting.

**Figure 1:**
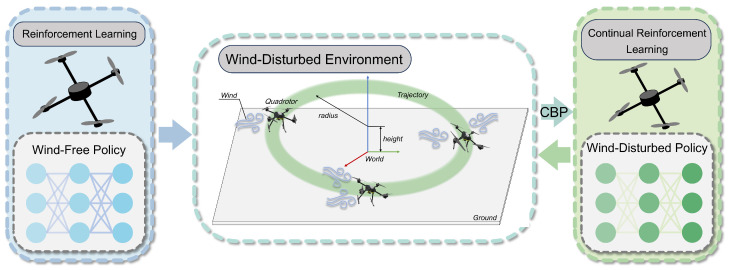
Overall architecture of the proposed continual reinforcement learning framework for quadrotor trajectory tracking under wind disturbance environment.

To address trajectory tracking control for quadrotors under significantly varying wind disturbances and mitigate catastrophic forgetting during continual learning, this paper proposes a continual reinforcement learning framework. This framework resolves the inability of RL policies to adapt to substantial environmental changes during quadrotor trajectory tracking in wind-disturbed environments. Most existing continual learning methods operate at either the parameter level [27,28] or the functional output level [29], focusing on global connection strength or output similarity. Continual backpropagation (CBP) [30] employs selective resetting of inefficient neurons at the structural level, continuously activating dormant units while maintaining an effective representation rank without additional loss terms, old model storage, or parameter importance evaluation, thereby enabling lightweight continual learning. As illustrated in Figure 1, our framework first trains a foundation trajectory tracking policy using proximal policy optimization (PPO) in wind-free conditions. Subsequently, it integrates CBP-based continual reinforcement learning to adapt this foundation policy to progressively evolving wind fields. By enhancing neural network plasticity through CBP, the framework improves quadrotor adaptation in dynamic environments. Additionally, a novel reward function is designed to enhance policy accuracy and effectiveness. In summary, this work investigates quadrotor trajectory tracking in disturbed environments via continual deep reinforcement learning. The primary contributions are summarized as follows:

1. We propose a novel continual reinforcement learning framework for quadrotor trajectory tracking, which significantly enhances adaptability in continuously varying wind fields.
2. We analyze the characteristics of tracking control and design an innovative reward function to accelerate network training convergence and improve control precision.
3. We validate the proposed framework on a quadrotor simulation platform. Experimental results demonstrate that our method achieves high trajectory tracking accuracy both in wind-free conditions and under gradually increasing wind disturbances, evidencing its strong learning capability.

The remainder of this paper is organized as follows. Section 2 formulates the trajectory tracking problem and elaborates on the reward function design. Section 3 introduces the proposed continual reinforcement learning framework and its core mechanisms. Section 4 presents comprehensive experimental results with comparative analysis under wind-disturbed conditions. Finally, Section 5 concludes this paper and discusses future research directions.

## 2. Preparation

### 2.1. Representation of System and Action

**Figure 2:**
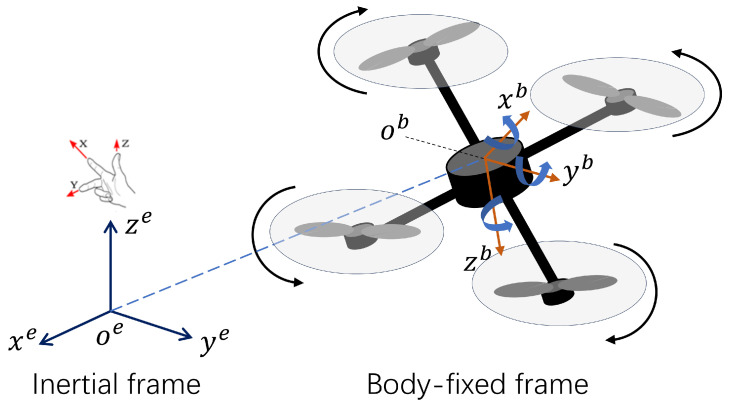
Structure illustration of a quadrotor.

The quadrotor is a canonical underactuated system capable of generating six-degree-of-freedom (6-DOF) motion through only four independent control inputs. The relationship between the quadrotor’s body frame and the inertial frame is illustrated in Figure 2. The time evolution of the quadrotor’s flight state can be described by differential equations. The position of the quadrotor in the inertial frame is denoted as $p^{e}={\left[p_{x},p_{y},p_{z}\right]}^{T}$, and ${\dot{p}}^{e}={\left[v_{x},v_{y},v_{z}\right]}^{T}\in R^{3}$ denotes the velocity vector expressed in the inertial frame. The quadrotor’s attitude is represented by Euler angles, whose time derivatives are governed by the kinematic relationship $\dot{\Theta }=W\cdot \omega^{b}\in R^{3}$ with

(1) $$\dot{\Theta }={\left[\dot{\alpha }\;\dot{\beta }\;\dot{\gamma }\right]}^{T},\;\omega^{b}={\left[\omega_{xb}\;\omega_{yb}\;\omega_{zb}\right]}^{T}$$

In Equation (1), Θ denotes the Euler angles, [αβγ] denotes roll, pitch, and yaw, respectively, and $\omega^{b}\in R^{3}$ represents the angular velocity in the body frame. The coordinate transformation matrix $W\in R^{3\times 3}$ is defined as follows:

(2) $$W=\left[\begin{array}{ccc} 1 & \tan\beta \;\sin\alpha & \tan\beta \;\cos\alpha \\ 0 & \cos\alpha & -\sin\alpha \\ 0 & \sin\alpha /\cos\beta & \cos\alpha /\cos\beta \end{array}\right]$$

This study designs a deep reinforcement learning-based control policy that enables the quadrotor to perform precise trajectory tracking tasks. In the tracking scenario, the motion relationship between the quadrotor and the reference trajectory is critical for controller design. We define a 9-dimensional state vector at time *t* as

(3) $$S_{t}^{*}={\left\{\Delta p_{x},\Delta p_{y},\Delta p_{z},\Delta \alpha ,\Delta \beta ,\Delta \gamma ,\Delta v_{x},\Delta v_{y},\Delta v_{z}\right\}}^{T}\in R^{9}$$

The state vector $S_{t}^{*}$ can be decomposed into three subset vectors. $\Delta p_{t}^{e}={\left[\Delta p_{x},\Delta p_{y},\Delta p_{z}\right]}^{T}$ represents the relative distance between the quadrotor’s current position and the target position in three coordinates. $\Delta \Theta_{t}={\left[\Delta \alpha ,\Delta \beta ,\Delta \gamma \right]}^{T}$ denotes the relative difference between the quadrotor’s Euler angles and the target attitude. The relative velocity between the quadrotor and the target is represented as $\Delta v_{t}^{e}={\left[\Delta v_{x},\Delta v_{y},\Delta v_{z}\right]}^{T}$. The dynamic behavior of the quadrotor over time, its motion trends, and the environmental context enable the neural network to better comprehend the system and generate effective control strategies for the quadrotor. Considering the kinematic relationship between the quadrotor and target states, this study defines the state observation for DRL as the current state concatenated with the states from the previous *n* timesteps, formulated as follows:

(4) $$S_{t}=\left\{S_{t}^{*},S_{t-1}^{*},S_{t-2}^{*},\ldots ,S_{t-n}^{*}\right\}\in R^{9(n+1)}$$

The position of the quadrotor can be controlled using its inertial-frame velocity components $v^{e}$ and yaw angle γ. Consequently, the action space for the reinforcement learning agent is defined as $u_{t}=\left[v_{x},v_{y},v_{z},\gamma \right]\in R^{4}$, where $\left\{v_{x},v_{y},v_{z}\right\}$ represent the inertial-frame velocities and γ denotes the yaw angle.

### 2.2. Design of Reward Function

A well-designed reward function facilitates faster convergence and superior performance during the reinforcement learning process. By analyzing the characteristics of trajectory tracking control tasks across different stages and integrating state observations, this study designs the reward function as follows:

(5) $$r_{t}=-(\omega_{1}∥\Delta p_{t}^{e}∥+\omega_{2}∥\Delta \Theta_{t}∥+\omega_{3}∥\Delta v_{t}^{e}∥+\omega_{4}∥u_{t}-u_{t-1}∥)+r_{p}^{+}+r_{crash}^{-}$$

Error terms related to the quadrotor’s state constitute penalty components. In Equation (5), $\left\{\omega_{1},\omega_{2},\omega_{3},\omega_{4}\right\}$ denote the weighting coefficients for the position penalty, attitude penalty, velocity penalty, and control smoothness penalty, respectively. Reward sparsity is a common challenge in reinforcement learning environments for quadrotor trajectory tracking tasks [31]. To address this, sparse reward terms $\left\{r_{p}^{+},r_{crash}^{-}\right\}$ are incorporated into the reward function. $r_{p}^{+}$ represents a positive sparse reward for positional accuracy, granted when $∥\Delta p_{t}^{e}∥\leq d$. $r_{crash}^{-}$ denotes a significant negative sparse penalty for critical failures (e.g., crashes or exceeding operational boundaries), serving to constrain abnormal quadrotor behavior. The inclusion of sparse rewards mitigates the agent’s over-reliance on local reward signals, thereby reducing the risk of overfitting and accelerating the learning process.

## 3. Continual Reinforcement Learning Method

### 3.1. Problem Description

In this study, environmental variations are formulated as a sequence of temporally ordered subtasks. The state transition function drifts during environmental changes while the state and action spaces remain invariant. Let $D=[E_{1},\ldots ,E_{h-1},E_{h},\ldots ]$ denote the dynamic environment set, where $E_{h}$ represents specific characteristics of the dynamic environment at the *h*-th stage. The primary objective of our reinforcement learning model is to derive an optimal control policy that maximizes the expected reward J(θ) under network parameters θ. The expected long-term return is expressed as follows:

(6) $$J\left(\theta \right)=E_{\tau ∼\pi_{\theta }\left(\tau \right)}\left[r\left(\tau \right)\right]=E_{\tau ∼\pi_{\theta }\left(\tau \right)}\left[\sum_{i=0}^{\infty }\zeta^{i}r_{i}\right]$$

where $\tau =\{s_{0},a_{0},s_{1},a_{1},\ldots \}$ denotes a complete episode trajectory, $r_{i}$ represents the instantaneous reward after agent–environment interaction, and ζ is the discount factor. According to the policy gradient theorem, the expectation of r(τ) under the distribution $\pi_{\theta }\left(\tau \right)$ is given by

(7) $$\begin{array}{c} \nabla J\left(\theta \right)=E_{\tau ∼\pi_{\theta }\left(\tau \right)}\left[r\left(\tau \right)\nabla \log\pi_{\theta }\left(\tau \right)\right] \end{array}$$

During the learning process, model parameters are optimized through policy approximation, expressed as follows:

(8) $$\theta_{t-1}^{*}=\arg\max_{\theta \in R^{d}}J_{E_{h-1}}\left(\pi_{\theta }\right)$$

The external disturbance problem refers to situations involving environmental transitions. The goal of continual learning is to enable autonomous updating from $\theta_{t-1}^{*}$ to $\theta_{t}^{*}$ when environmental characteristics transition to $E_{h}$:

(9) $$\theta_{t}^{*}=\arg\max_{\theta \in R^{d}}J_{E_{h}}\left(\pi_{\theta }\right)$$

### 3.2. The PPO Algorithm

**Figure 3:**
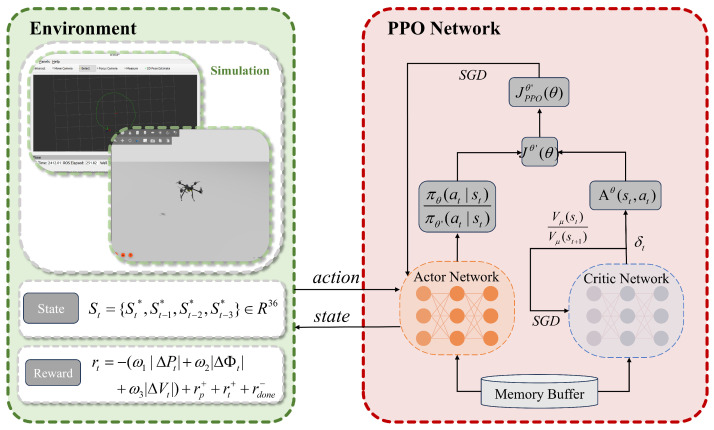
Quadrotor trajectory tracking control based on PPO.

Compared to value-based methods, policy-based reinforcement learning offers distinct advantages by directly learning the policy itself, thereby accelerating the learning process and enhancing decision-making performance in continuous action spaces. PPO integrates policy gradients with importance sampling to address the inherent sample inefficiency of basic policy gradient methods. Utilizing an actor–critic architecture, the PPO-based quadrotor controller design is illustrated in Figure 3.

PPO employs generalized advantage estimation (GAE), which balances the trade-offs between Monte Carlo methods and single-step temporal difference (TD) approaches through weighted fusion of multi-step TD errors. The GAE formulation is defined as follows:

(10) $$A^{\theta }\left(s_{t}|a_{t}\right)=A_{GAE}^{\theta }=\sum_{l=0}^{T-t}{\left(\zeta \lambda \right)}^{l}\delta_{t+l},\;\delta_{t}=r_{t}+\zeta V\left(s_{t+1}\right)-V\left(s_{t}\right)$$

In Equation (10), λ serves as a trade-off parameter regulating the weighting distribution of multi-step returns, $\delta_{t}$ denotes the single-step TD error, and $V(s_{t})$ represents the state-value function. This design enables the advantage function to capture global information from multi-step interactions while mitigating the high variance inherent in Monte Carlo methods.

To enhance policy update stability and prevent excessive fluctuations that could destabilize optimization, the algorithm incorporates the advantage function $A^{\theta }\left(s_{t},a_{t}\right)$ and importance sampling into the objective function (6), yielding the surrogate objective function:

(11) $$J^{\theta^{\prime }}\left(\theta \right)=E_{\tau ∼\pi_{\theta }\left(\tau \right)}\left[\frac{\pi_{\theta }\left(a_{t}|s_{t}\right)}{\pi_{\theta^{\prime }}\left(a_{t}|s_{t}\right)}A^{\theta^{\prime }}\left(s_{t}|a_{t}\right)\right]$$

In Equation (11), $\theta^{\prime }$ denotes the behavioral policy parameters interacting with the environment, while θ represents the optimization parameters. The objective $J^{\theta^{\prime }}\left(\theta \right)$ is computed via the product of the advantage $A^{\theta }\left(s_{t},a_{t}\right)$ (evaluated from samples $s_{t},a_{t}$) and the tractable importance ratio $\frac{\pi_{\theta }\left(a_{t}|s_{t}\right)}{\pi_{\theta^{\prime }}\left(a_{t}|s_{t}\right)}$. To maintain proximity between the behavioral policy $\pi_{\theta^{\prime }}\left(a_{t}|s_{t}\right)$ and optimized policy $\pi_{\theta }\left(a_{t}|s_{t}\right)$ during importance sampling, we enforce policy constraints through a clipping function, resulting in the final objective:

(12) $$J_{PPO}^{\theta^{\prime }}\left(\theta \right)=\sum_{\left(s_{t},a_{t}\right)}\min\left(\frac{\pi_{\theta }\left(a_{t}|s_{t}\right)}{\pi_{\theta^{\prime }}\left(a_{t}|s_{t}\right)}A^{\theta^{\prime }}\left(s_{t},a_{t}\right),\mathrm{clip}\left(\frac{\pi_{\theta }\left(a_{t}|s_{t}\right)}{\pi_{\theta^{\prime }}\left(a_{t}|s_{t}\right)},1-\epsilon ,1+\epsilon \right)A^{\theta^{\prime }}\left(s_{t},a_{t}\right)\right)$$

By integrating generalized advantage estimation and a clipping mechanism, PPO effectively balances bias–variance trade-offs during policy optimization while preventing abrupt policy updates. These properties establish PPO as an ideal solution for trajectory tracking in quadrotors, particularly when addressing continuous action spaces and multiple disturbance scenarios.

### 3.3. CBP Method

**Figure 4:**
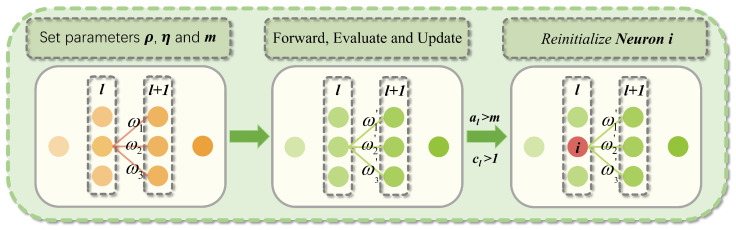
Continual backpropagation mechanism.

In dynamic environments, quadrotors must continuously adapt to fluctuating wind conditions. While traditional deep learning approaches perform well in static settings, they suffer from loss of plasticity in continual learning scenarios [30]. To enhance the agent’s adaptability to dynamic environments and facilitate efficient exploration, this study integrates continual backpropagation (CBP) into the PPO framework. Unlike conventional approaches that freeze network weights after training, CBP reinitializes neurons at the structural level to preserve architectural diversity. Figure 4 illustrates the operational principle of CBP within the neural network architecture.

The principal advantage of CBP lies in its unit utility assessment and selective reinitialization mechanism. The utility metric for hidden unit *i* in layer *l* quantifies its contribution to downstream layers via an exponentially weighted moving average:

(13) $$u_{i}^{l}=\eta \cdot u_{i}^{l}+\left(1-\eta \right)\cdot |h_{i,t}^{l}|\cdot \sum_{k=1}^{n_{l+1}}\left|w_{i,k}^{l}\right|$$

where η denotes the decay rate balancing historical and current contributions, $h_{i,t}^{l}$ represents the activation of unit *i* in layer *l* at time *t*, and $w_{i,k}^{l}$ denotes the weight connecting unit *i* in layer *l* to unit *k* in layer l+1.

When resetting a hidden unit, CBP initializes all outgoing weights to zero to prevent perturbation of learned network functions. However, this zero-initialization renders new units immediately nonfunctional and vulnerable to premature resetting. To mitigate this, newly initialized units receive a grace period of *m* updates during which they are reset-exempt. Only after exceeding this maturity threshold *m* are units considered mature. Subsequently, at each update step, a fraction ρ of mature units per layer undergo reinitialization. In practice, ρ is set to an extremely small value, ensuring approximately one unit replacement per several hundred updates.

During each network parameter update, CBP introduces only two additional operations compared to standard forward/backward propagation: unit utility updates and neuron resets. The unit utility update applies an exponential moving average to every neuron in each hidden layer, involving one absolute-value summation and one multiply–accumulate per weight. Its time complexity is $O\left(\sum_{l}n_{l}\cdot n_{l+1}\right)$, which is on the same order as the total number of network parameters. Neuron resets select and reinitialize a constant number of mature units at a very low rate ρ; because this occurs infrequently and affects only a fixed number of units per event, its overall overhead is negligible. In terms of memory overhead, CBP allocates for each hidden unit an additional utility value $u_{i}^{l}$ and age $a_{i}^{l}$, totaling $O\left(\sum_{l}n_{l}\right)$. Thus, CBP maintains continuous structural variation and network plasticity with minimal extra time and space costs, fully compatible with standard neural network training pipelines.

Through CBP integration, the quadrotor UAV iteratively updates network parameters to maintain adaptability in complex, dynamically evolving environments.

### 3.4. Integrated Method

**Figure 5:**
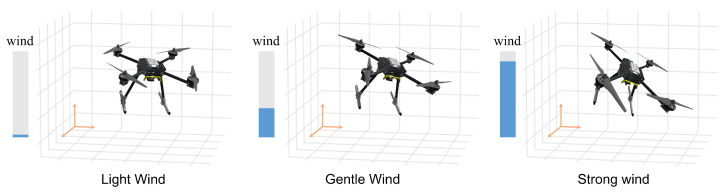
Quadrotor flight behavior under different wind conditions.

Reinforcement learning experiments exhibit greater stochasticity and variance compared to supervised learning due to inherent algorithmic randomness and sequential data dependencies influenced by the learning process itself [32,33]. Figure 5 illustrates quadrotor flight dynamics under varying wind conditions: stable flight occurs under low winds; moderate winds induce attitude deviations and instability; and high winds cause significant flight disruption or failure.

A foundation policy $\pi_{\theta }$ trained in environment $E_{h-1}$ exhibits degraded performance when deployed in altered environment $E_{h}$, necessitating adaptive capability. This study proposes a continual reinforcement learning framework where wind-free control strategies serve as foundational models, enabling policy adaptation to environmental changes. By integrating CBP’s continual learning mechanism into PPO’s policy gradient updates, our approach maintains learned policy retention while flexibly generating new action distributions, achieving rapid adaptation to dynamic wind fields.

Algorithm 1 details the continual reinforcement learning process for updating quadrotor control policy $\pi_{\theta }$ in dynamic environments. Wind disturbance signals serve as environmental variables during trajectory tracking. The policy $\pi_{\theta }$ pretrained in $E_{h-1}$ serves as the foundation model, where the agent learns through standard policy gradients in $E_{h}$ while CBP concurrently performs structural neuron updates. After each full network update, neurons meeting reinitialization criteria are reset. Since the agent has acquired fundamental representations of the $E_{h-1}$ state–action space, the CBP algorithm enhances network plasticity to facilitate adaptation to $E_{h}$ variations.
**Algorithm 1:** Continual reinforcement learning for UAV control. 
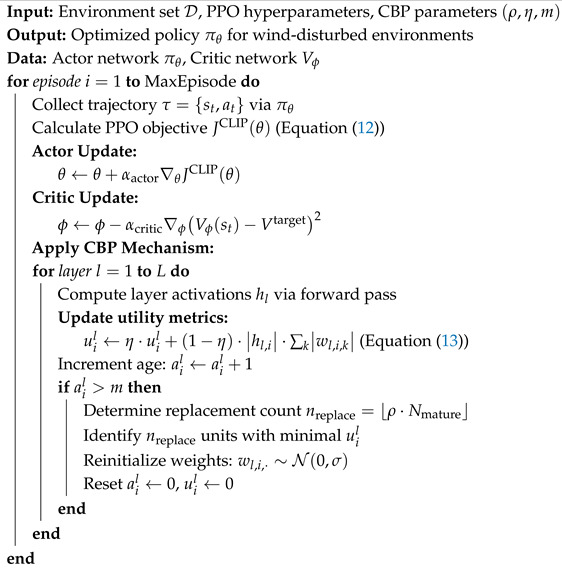


## 4. Experiment and Discussion

### 4.1. Experimental Setup and Parameter Configuration

The training process was executed on an Ubuntu 22.04 system equipped with an AMD Ryzen 7 6800H CPU and NVIDIA RTX 3050 GPU. Our continual reinforcement learning control algorithm was implemented in Python 3.10 using PyTorch 2.4, with the Adam optimizer employed for network training [34]. We established a simulation environment integrating Gazebo 2 with the PX4 flight controller, leveraging Gazebo’s high-fidelity physics engine to simulate quadrotor flight under wind disturbances [35]. The parameters of the quadrotor are shown in Table 1. The learning framework follows the architecture depicted in Figure 1.

The PPO implementation utilizes separate actor and critic networks, each containing four hidden layers. The actor network processes state observations $S_{t}\in R^{36}$ as input and outputs system actions $u_{t}\in R^{4}$, with hidden layer dimensions of 64, 128, 256, and 64 neurons, respectively. The critic network accepts system states and actions as input and estimates state value $V(S_{t})$, featuring hidden layers of 128, 256, 256, and 128 neurons. All hidden layers employ ReLU activation functions.

Reward function weights were empirically determined, with state error components defined as penalty terms to promote stable flight control. Given the critical importance of position and attitude control in trajectory tracking, larger weights were assigned to position ($\omega_{1}=0.12$) and attitude ($\omega_{2}=0.12$) penalties, while velocity and control smoothness penalties received lower weights ($\omega_{3}=0.05$, $\omega_{4}=0.1$). To enhance exploration and simplify training, two sparse reward components were implemented: a positional accuracy reward $r_{p}^{+}=9$ triggered when $∥\Delta p_{t}^{e}∥<d=0.05\;m$ and a critical failure penalty $r_{crash}^{-}=-100$ imposed for crashes or boundary violations.

The operational boundary for trajectory tracking was defined as a 5m×5m×5m cubic volume. Additional training parameters, referenced from prior works [25,36], are detailed in Table 2.

To evaluate the performance of the continual reinforcement learning-based quadrotor control strategy in windy environments, this study conducts trajectory tracking experiments under both wind-free and wind-disturbed conditions. The root mean square error (RMSE) of tracking deviation serves as the quantitative evaluation metric for assessing the tracking control performance of the proposed method. Let *M* denote the total number of samples and $e_{i}$ represent the positional tracking error of the *i*-th sample. The RMSE is calculated as follows:

(14) $$\mathrm{RMSE}=\sqrt{\frac{1}{M}\sum_{i=1}^{M}e_{i}^{2}}\;\forall i\in A$$

### 4.2. Performance Analysis in the Wind-Free Environment

**Figure 6:**
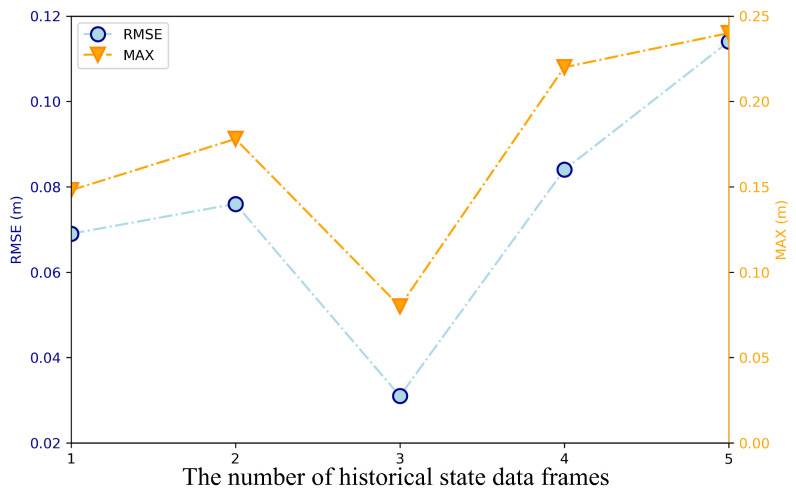
Comparison of the quadrotor’s position between the reference and the predictions.

The reward function design incorporates not only the current flight state but also historical states from the preceding *n* timesteps to capture motion trends and environmental context for policy learning. Figure 6 illustrates the variation in control error RMSE and maximum error with different history lengths *n*. The curves demonstrate significant error reduction as *n* increases from 0 to 3. For n>3, errors exhibit a slight increase, indicating that excessive historical information introduces redundant noise. Consequently, this study selects n=3 as an optimal compromise between information sufficiency and network complexity.

**Figure 7:**
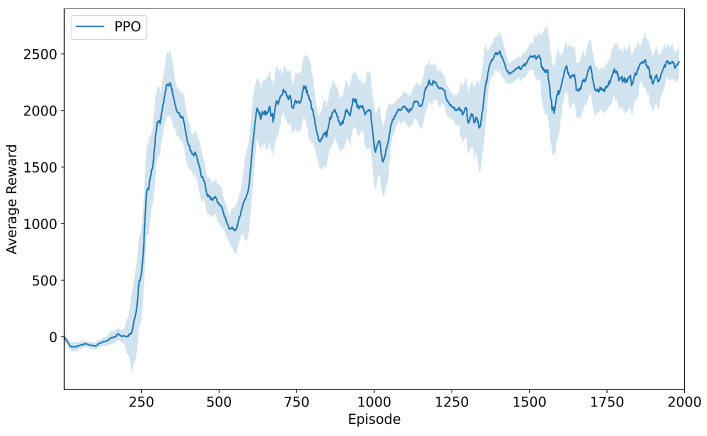
Training reward progression under wind-free conditions.

Under wind-free conditions, we trained the quadrotor’s trajectory tracking policy using the PPO algorithm over 2000 training episodes. Figure 7 shows the corresponding average reward curve: During the initial 200 episodes, the policy engaged primarily in random exploration, resulting in substantial reward fluctuations near zero. Between episodes 200 and 350, guided by the advantage function, the policy distribution converged toward optimal trajectories, causing rapid reward growth. From episodes 350 to 600, the clipped probability mechanism constrained excessive gradient updates, leading to a transient reward decrease. Beyond episode 600, entropy regularization stabilized the reward curve, maintaining high values with minimal oscillations.

**Figure 8:**
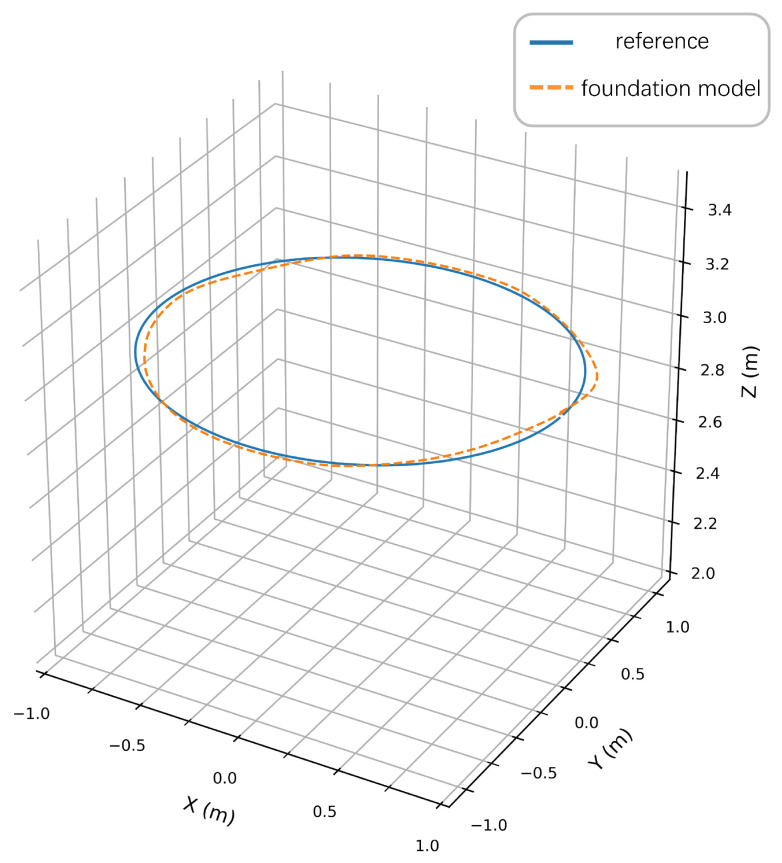
Reference versus actual trajectory in wind-free environment.

**Figure 9:**
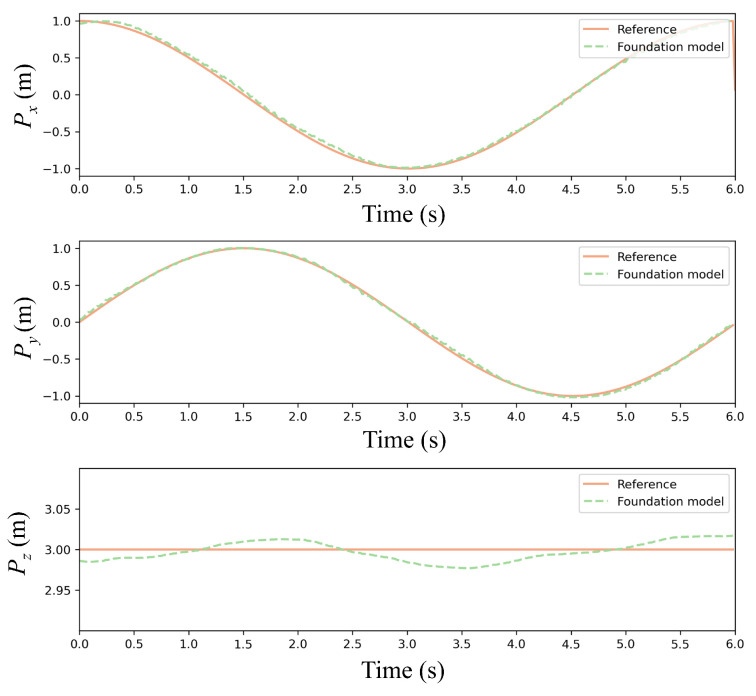
Comparison of the results of the foundation model and reference trajectory.

**Figure 10:**
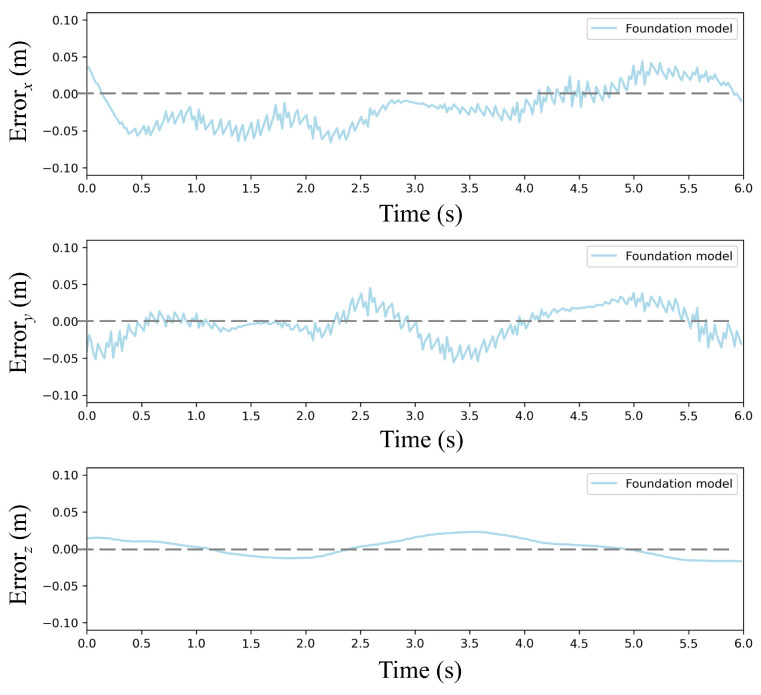
Error of the foundation model.

To quantitatively validate the pretrained PPO policy, Figure 8 compares reference and actual trajectories in a disturbance-free environment. The quadrotor tracked a circular path with radius = 1 m from initial position (1, 0, 3) m at a constant linear velocity (1 m/s). As shown in Figure 8, the 3D trajectory plot explicitly compares the reference and prediction with the foundation model. As demonstrated in Figure 9 and Figure 10, the foundation model achieves high-precision trajectory tracking in wind-free environments. The policy achieves precise tracking with near-perfect circular closure, indicating negligible cumulative drift. Maximum deviations occur at curvature maxima where centrifugal forces peak, yet all-axis errors remain below 0.05 m, satisfying precision thresholds for quadrotor maneuvering tasks.

This result empirically verifies that the PPO-derived policy converges to a robust nominal controller under ideal conditions, establishing a foundational model for subsequent continual learning.

### 4.3. Performance Analysis in the Wind-Disturbed Environment

#### 4.3.1. Performance in Stepwise Wind

**Figure 11:**
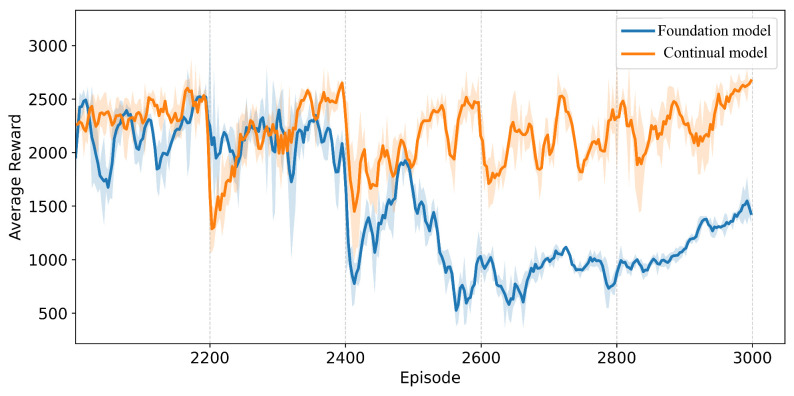
Average reward comparison under stepwise wind disturbances.

To evaluate the continual learning capability of our algorithm in dynamic wind fields, we constructed a stepwise wind variation scenario: after 2000 episodes of foundation model training, a wind speed increment of 7 m/s was applied every 200 episodes along both *x*- and *y*-axes, creating a progressively intensifying wind field. Figure 11 compares the average reward curves of PPO and our continual reinforcement learning approach, with the horizontal axis representing training episodes and the vertical axis the average reward. Experimental results demonstrate that during low-wind phases, both methods exhibit comparable oscillation amplitudes while maintaining high returns. As wind speed progressively increases, the PPO strategy shows continuous reward degradation, indicating insufficient disturbance adaptability. In contrast, the CBP-enhanced policy exhibits transient reward drops after each wind speed change but rapidly recovers to prior levels, maintaining stable oscillations thereafter. The characteristic sawtooth reward pattern signifies substantially enhanced network plasticity, confirming the algorithm’s continual learning and rapid adaptation capabilities in dynamic environments.

**Figure 12:**
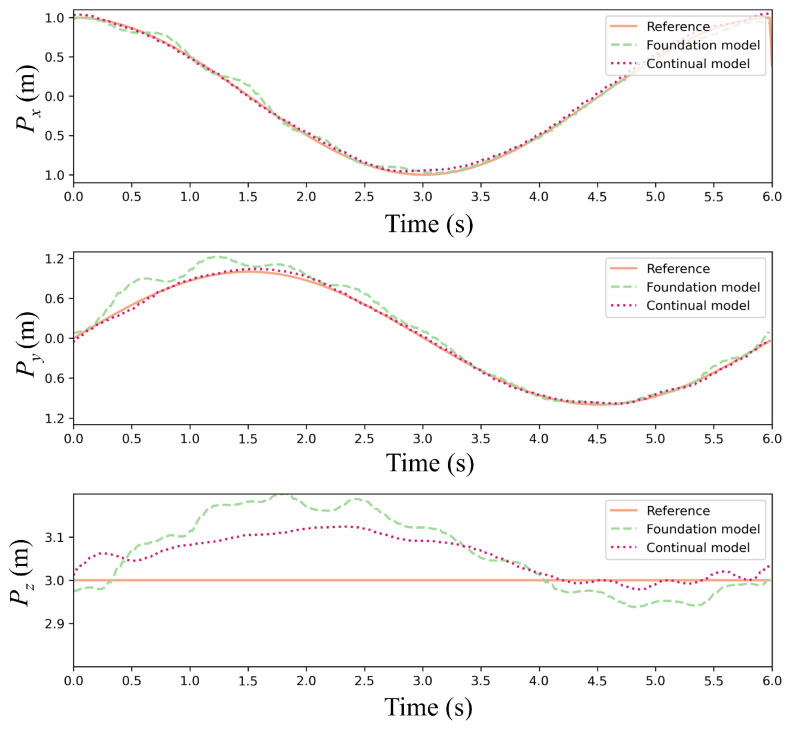
Trajectory comparison of the foundation model and continual model under stepwise wind disturbances.

**Figure 13:**
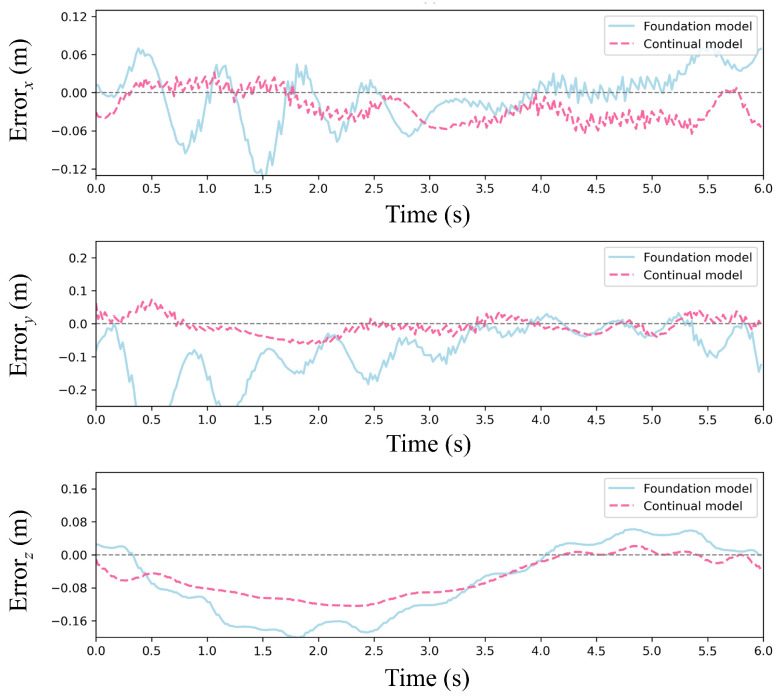
Error of the foundation model and continual model under stepwise wind disturbances.

Figure 12 and Figure 13 present trajectory comparisons under extreme wind conditions (cumulative 35 m/s along *x*/*y*-axes). Figure 12 displays reference versus controlled trajectories in three spatial dimensions, while Figure 13 shows corresponding positional errors. The results indicate that our continual reinforcement learning achieves lower error peaks than PPO at each wind speed increment while maintaining faster recovery to foundation performance. Overall average error is reduced by approximately 15%, validating the CBP algorithm’s significant enhancement of policy robustness and precision. Critically, the continual reinforcement learning method maintains accurate path tracking under strong disturbances, whereas standard PPO exhibits substantial deviation.

These findings demonstrate that CBP’s selective neuron resetting and structural diversity preservation empower reinforcement learning policies to maintain efficacy in evolving wind fields, significantly enhancing quadrotor adaptation to novel conditions.

#### 4.3.2. Performance Under Stochastic Wind

**Figure 14:**
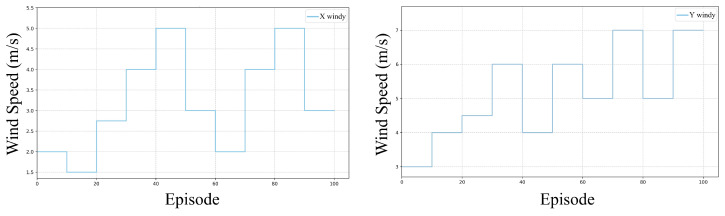
Stochastic wind speed variations.

To validate the adaptability of the proposed continual reinforcement learning framework in realistic wind environments, we conducted additional trajectory tracking experiments in a stochastic wind scenario. As depicted in Figure 14, random sampling of wind speeds in the *x*-axis $v_{w,x}$ and *y*-axis $v_{w,y}$ was performed every 10 episodes (approximately 1 min). Both $v_{w,x}$ and $v_{w,y}$ were subject to a normal disturbance distribution with parameters N(4,2.5). The trajectory reference entailed a circular path with a radius of 1 m starting from the initial position at (1, 0, 3) m, moving at a constant linear velocity of 1 m/s. The evaluation criterion remained the RMSE of positional tracking, comparing two methods: the foundational model and the proposed continual reinforcement learning model. The training duration spanned 100 episodes to observe long-term adaptation trends.

**Figure 15:**
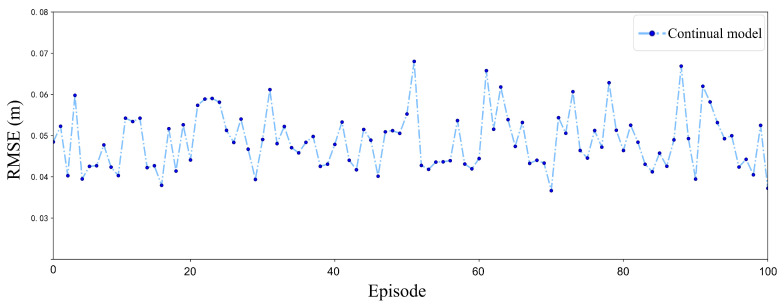
RMSE of continual model under stochastic wind disturbances.

Figure 15 illustrates the RMSE of the continual model training in a stochastic wind field over 100 episodes. The continual model exhibits notable stability throughout the training period. The RMSE values consistently remain low, predominantly below 0.06 m, indicating the efficacy of the CBP mechanism. This mechanism’s capacity to dynamically reset inefficient neurons enables the model to adjust to varying stochastic wind conditions without experiencing catastrophic forgetting. Consequently, when confronted with new wind patterns, the model promptly reconfigures its neural network to identify fresh optimal policies while retaining valuable insights from prior wind conditions.

**Figure 16:**
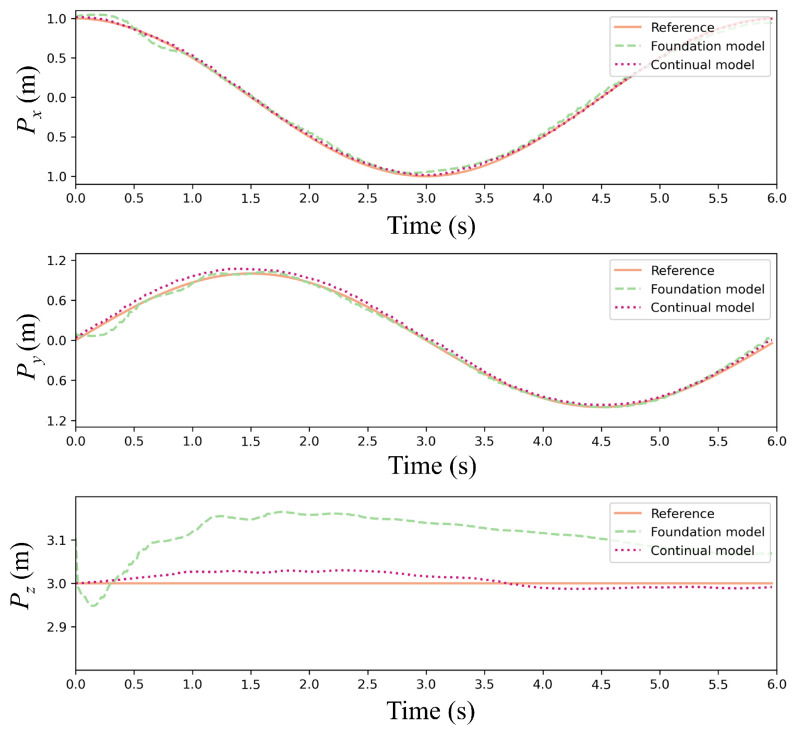
Trajectory comparison between the foundation model and continual model under stochastic wind disturbances.

**Figure 17:**
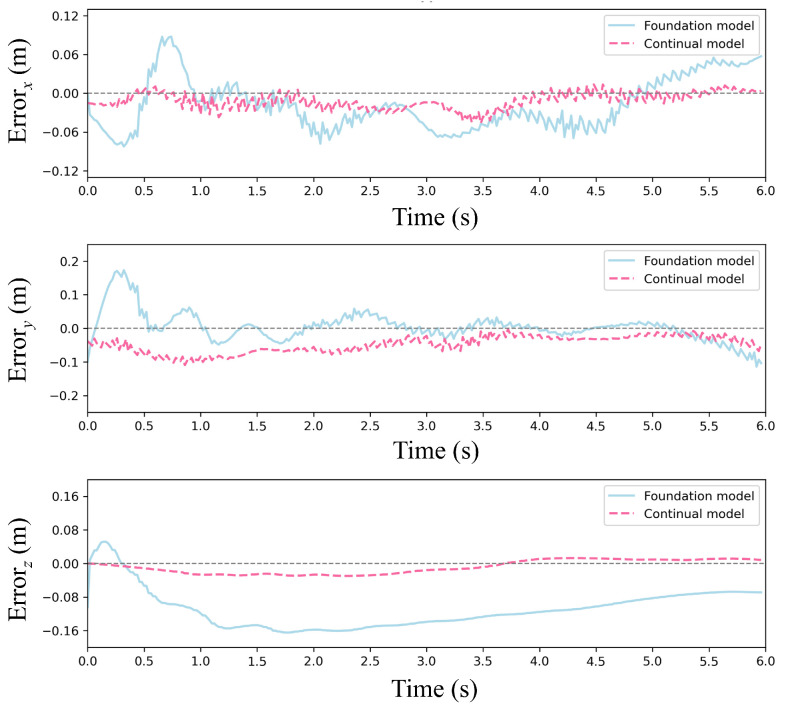
Error of the foundation model and continual model under stochastic wind disturbances.

Figure 16 and Figure 17 present a comparative analysis of the foundation model and the continual model in a single episode under random wind conditions. Figure 16 depicts trajectory comparisons between the two models, showing that the continual model closely aligns with the reference trajectory in the three directions, even amidst significant random wind disturbances. Further insights from Figure 17, focusing on error dynamics, reveal that the continual model maintains positional errors within ±0.06 m across all three directions, swiftly recovering from transient wind gusts. In contrast, the foundation model exhibits persistent error accumulation, particularly evident in y-direction errors exceeding ±0.2 m in later episodes. This disparity arises from the utility-based neuron management of CBP, wherein underperforming neurons are reset to explore adaptive strategies in response to new disturbance patterns induced by random winds while retaining valuable features learned from prior wind conditions.

Stochastic wind disturbances emulate natural atmospheric conditions more accurately, featuring unstructured variations in magnitude and direction. This necessitates enhanced real-time adaptation and robustness from the controller. The outcomes in the stochastic scenario validate the superior performance of the suggested framework over the conventional PPO model in terms of real-time adaptability and robustness.

## 5. Conclusions

This study establishes a high-precision quadrotor trajectory tracking foundation model based on the proximal policy optimization algorithm and then introduces the continual backpropagation algorithm to propose a continual reinforcement learning framework for dynamic wind-disturbed environments. Simulation results demonstrate that compared to standard PPO, our continual reinforcement learning approach achieves lower tracking errors under multiple wind speed increments. These findings validate the significant enhancement of policy precision and robustness through continual learning mechanisms. Future work will explore CBP applications in real-flight validation, cross-task transfer learning, and more complex mission scenarios to enhance the generalization and cross-task adaptation capabilities of UAV reinforcement learning strategies.

## Figures and Tables

**Table 1:** Parameters of the quadrotor.

Parameter	Value
Mass of the quadrotor	2 kg
Principal moment of inertia	$\left(0.0217,\;0.0217,\;0.04\right)\;\mathrm{kg}\cdot m^{2}$
Motor constant	$8.549\times 10^{-6}\;\mathrm{kg}\cdot m$
Moment constant	$0.016\;\mathrm{kg}\cdot m^{2}$
Wheelbase	0.348m

**Table 2:** Training parameters.

Parameter	Value
GAE bias–variance (λ)	0.95
Actor learning rate	$2\times 10^{-4}$
Critic learning rate	$3\times 10^{-4}$
Policy update epochs	9
Initial action standard deviation	0.4
Action std decay rate	$2\times 10^{-4}$
Learning rate decay coefficient	$10^{-4}$
Discount factor (ζ)	0.98
CBP replacement rate (ρ)	$10^{-5}$
CBP utility decay rate (η)	0.99
CBP maturity threshold (*m*)	50

## Data Availability

The original contributions presented in this study are included in the article; further inquiries can be directed to the corresponding author.
